# Annotations

**Published:** 1889-06-29

**Authors:** 


### Annotations.

**Monkeys as "Topers."**

That many persons, both male and female, are constitutionally predisposed to the liking for strong liquors is a fact highly probable in itself, and confirmed by experience and by the analogous cases of some of the lower animals. The experiences of scientists, who have compared the tastes and behaviour of certain monkeys with those of man under similar conditions, have sometimes been highly ridiculous. "Many kinds of monkeys," says Darwin (*Descent of Man*) "have a strong taste for tea, coffee, and spirituous liquors. They will also, as I have myself seen, smoke tobacco with pleasure. Brehm asserts that the natives of North-Eastern Africa catch the wild baboons by exposing vessels with strong beer, by which they are made drunk. He has seen some of these animals, which he kept in confinement, in this state; and he gives a laughable account of their behaviour and strange grimaces. On following morning they were very cross and dismal; they held their aching heads with both hands, and wore a most pitiable expression. When wine or beer were offered to them they turned away with disgust, but relished the juice of lemons. An American monkey, an Ateles, after getting drunk on brandy, would never touch it again, and was thus," adds Darwin, "much wiser than men." It is plain that so far as the taste and immediate effects of stimulants are concerned, those animals, which so closely resemble man, have a natural liking for them. There is in human beings very often not only a similar liking, but, as the result of depressing circumstances, a diseased craving which it is exceedingly difficult to resist. It is necessary to recognise this natural craving, and to take into account the depression of mind which, when associated with it, often impels the victim to acts of drunkenness. Not only must these predisposing circumstances be taken into account in estimating the moral turpitude of any given inebriate, but also in considering his case with a view to cure. There is no doubt that in most cases of chronic alcoholism the most rigid application of the principle of total abstinence is called for. It need not be inferred that monkeys have either stronger intellects or better morals than men, because some who had once been made drunk by alcohol refused to taste it again. They were not chronic tipplers, and, therefore, had not acquired a morbid and imperious craving. For ordinary persons, it is enough to inculcate habits of temperance and moderation in eating as well as drinking; but in all cases of pronounced or incipient alcoholism, intoxicating drink of every kind should be strictly excluded, not for one or two years, but for life.

**How Children are Poisoned.**

The rearing of young children who have for any reason been deprived of mother's milk is a much more difficult and anxious business than it is generally supposed to be. Dr. Victor Vaughan, not long ago, discovered a poison which is produced in cow's milk under certain circumstances, and which is of a most deadly character. That poison he called "tyrotoxicon." It is developed in milk by the action of an organised ferment, in other words by a member of the notorious "bacterium" family. Tyrotoxicon was first found in ice-cream and in cheese that had produced violent symptoms of poisoning. Since that time careful experiments have been carried out by competent chemists, who have succeeded in isolating the poison and in demonstrating its kinship with the bacterial order. This deadly poison is by no means rarely developed; on the contrary, a very simple set of ordinary circumstances may give rise to it. The three main features that excite it to dangerous activity in milk, for example, are uncleanliness, the want of fresh air, and a temperature of 98deg. to 100deg. F. Many farm dairies and town pantries are so situated as to be always insufficiently supplied with fresh air. For several months in the summer also they are during many hours of the day subject to a temperature which often reaches 98deg. All that is wanted in these circumstances is some carelessness on the part of the kitchen-maid or mother, in washing, drying, and putting away dishes and bowls that are used for milk, and then all the conditions necessary for the development of this dangerous poison are present in full measure. The symptoms produced by tyrotoxicon, even when taken in small doses, are active vomiting and purging. If much of the milk containing the poison has been drunk, these symptoms are greatly intensified and a choleraic condition ensues. This frequently ends in rapid prostration and death. It is not desirable that mothers and nurses should be unnecessarily alarmed, as if some new poison had been invented, or created or developed, which had no previous existence. The tyrotoxicon which has so recently been isolated, classified, and made a part of knowledge, has always existed in association with milk which has been allowed to decompose or "turn sour" in the presence of dirt and summer heat. The merit of Dr. Victor Vaughan and his chemical friends consists in this, that they have strictly defined and made evident the real nature of the dangerous element in putrefying milk, and the conditions under which it developes. Mothers and nurses who take the trouble to read and understand the facts here set forth may absolutely prevent any risk whatever of injury or death from tyrotoxicon. All that is necessary is to keep their dairies, pantries, storerooms, and milk vessels scrupulously clean, to so arrange that a constant and abundant current of fresh air shall pass through the pantry or dairy, and to use ice or some other means of lowering the temperature in hot weather. Two other precautions are desirable—viz., to boil or, still better, stew the milk, and not to keep it too long before it is used. It is well to remember that not only whole milk, but articles which contain milk as a constituent, such as ice-cream, cheese and butter, may also give origin to the same poison and to the same set of dangerous symptoms in those who use these substances as food.

**The Value of Life.**

Some years ago the *Daily Telegraph* indulged in the phrase "The Value of Life and the Sacredness of Property," which certainly would give the impression that a prominent English newspaper, the organ *par excellence* of the middle classes, rated property higher than life. Probably, the phrase came from an unthinking writer with more ear for sound than eye for proportion; but near Salerno there has existed for years a band of thieves and murderers who have held life very cheap indeed. Not their own lives, however, after the fashion of the highwaymen of our youthful romances, but those of others. They would murder anybody if they were paid twenty francs for it—a human life was worth to them just 16s. 8d. They did not want the incentive of hatred or revenge, or even cupidity. With them murder was not, perhaps, a fine art, but an ordinary business. The captain of the band has, however, now been arrested, and is under sentence of death. Will he estimate his own life at just 16s. 8d.? Looking upon it from the point of view of the average citizen, we are inclined to ask if there is indeed no fundamental difference between ourselves and those human brutes who can kill their fellow-men so lightly. We would not boast of our virtue. It would not become any of us to say assuredly that, under some sudden impulse of passion, we would not commit murder, but, at least, we would not, in cold blood, kill a man against whom we cherished no malice. Is this a matter of constitution or education, as our modern scientists would tell us, a thing depending on the convolutions of the brain and certain habits of thought inherited from our ancestors? or does it depend on a thing which old-fashioned people called "the grace of God in our souls"? Here is your chance, scientists, who believe that all things have a physical origin, whose analytical minds will see nothing beyond what the knife will cut and the microscope unveil. This murderer Mottola will soon be dead. You are fond of examining brains—may it be forgiven you, if you also, in your scientific curiosity, hold life somewhat cheaply! Examine this man's brains, then, and point out to us the exact spot, the defect or excess of substance, which made it possible for him to become a murderer by habit and trade. Then we will believe that your theories cover all the phenomena of life—the growth of thought, the instincts of love and hate, as well as the processes of digestion. Till then we reserve our judgment, and not set down our ancestors as foolish or unscientific because they believed in a Supreme Power, and prayed to be delivered from the temptation of the devil.

